# Anatomical basis for sensory preservation in robotic mastectomy

**DOI:** 10.1093/bjs/znaf232

**Published:** 2025-11-18

**Authors:** Hunter A Holley, Sandra Hembrecht, Níamh M Smyth, Mathieu Colbert, Fabio Quondamatteo, Arnold D K Hill

**Affiliations:** Department of Graduate Entry Medicine, RCSI University of Medicine and Health Sciences, Dublin, Ireland; Department of Anatomy and Regenerative Medicine, RCSI University of Medicine and Health Sciences, Dublin, Ireland; Department of Surgery, RCSI University of Medicine and Health Sciences, Dublin, Ireland; Department of Surgery, Beaumont Hospital, Dublin, Ireland; Department of Surgery, RCSI University of Medicine and Health Sciences, Dublin, Ireland; Department of Surgery, Connolly Hospital Blanchardstown, Dublin, Ireland; Department of Graduate Entry Medicine, RCSI University of Medicine and Health Sciences, Dublin, Ireland; Department of Anatomy and Regenerative Medicine, RCSI University of Medicine and Health Sciences, Dublin, Ireland; Department of Surgery, RCSI University of Medicine and Health Sciences, Dublin, Ireland; Department of Surgery, Beaumont Hospital, Dublin, Ireland

## Abstract

**Background:**

Sensory preservation of the nipple-areolar complex (NAC) is crucial for physical, psychological, and sexual health after mastectomy. Robotic-assisted nipple-sparing mastectomy (rNSM) techniques have shown promise in preserving NAC sensation, but there is limited detailed anatomical evidence supporting this observation. The aim of this study was to characterize the anatomical pathways and variability of sensory nerves innervating the NAC, particularly focusing on implications for the development of improved nerve-preserving techniques during breast surgery.

**Methods:**

A cadaveric anatomical study on three adult female cadaveric donors (six breasts) was undertaken, which was complemented by a systematic review of the existing literature using a modified PRISMA approach.

**Results:**

The anterior cutaneous branch (ACB) and the lateral cutaneous branch (LCB) of the fourth intercostal nerve (ICN) were consistently the primary cutaneous nerves innervating the NAC. The ACB of the fourth ICN, particularly its lateral division, followed a superficial and consistent subdermal route bypassing breast tissue in all cadavers. Conversely, the LCB of the fourth ICN, specifically its anterior division, traversed deeper breast tissue to reach the NAC. Variable supplementary contributions were observed from the second, third, and fifth ICNs, and previously undocumented ancillary branches. Significant inter-individual anatomical variability was noted.

**Conclusion:**

The consistent superficial pathway of the ACB of the fourth ICN provides a clear anatomical rationale for improved sensory preservation observed in rNSM, given the procedure’s lateral incision and precise dissection capabilities. Recognition of anatomical variability and detailed nerve trajectories should guide surgical planning to optimize sensory outcomes in breast cancer surgery and reconstruction.

## Teaser

This cadaveric and systematic review study characterizes the sensory innervation of the nipple-areolar complex (NAC), identifying the anterior cutaneous branch of the fourth intercostal nerve as a consistent, superficial contributor to NAC sensation. These findings offer anatomical support for improved sensory outcomes observed in robotic-assisted nipple-sparing mastectomy and highlight the importance of nerve-preserving strategies in breast cancer surgery and reconstruction.

## Introduction

The nipple-areolar complex (NAC) plays a critical role in lactation, sexual function, and psychological well-being, hence the importance of sensory-preserving or -restoring techniques for optimizing post-mastectomy quality of life. The innervation of the NAC is primarily from the third, fourth, and fifth intercostal nerves (ICNs), with prior anatomical studies identifying the branches of the fourth ICN as the dominant contributors to NAC sensation^1^. The anterior aspect of the lateral cutaneous branch (LCB) of the fourth ICN has frequently been cited as the most consistent nerve supply to the NAC^2,3^. However, multiple nerves contribute to NAC sensation, including the anterior cutaneous branches (ACBs) of the third, fourth, and fifth ICNs, with significant variability. A recent meta-analysis found the LCB of the fourth ICN to contribute in 89% of cases, and the ACBs of the third and fourth ICNs to contribute in 54% and 68% of cases respectively, reflecting a variable, multi-nerve innervation pattern^2^. Despite clinical significance, detailed pathways of the sensory innervation of the NAC remain poorly characterized in the literature. This knowledge gap has direct clinical implications, especially as emerging robotic-assisted nipple-sparing mastectomy (rNSM) techniques increasingly demonstrate the possibility of improved preservation of NAC sensation.

The breast develops from the mammary ridge during the fifth gestational week, with subsequent contributions from ectodermal and mesenchymal tissues forming the vascular, glandular, and connective tissues^4^. This development results in a relatively consistent segmental arrangement of ICNs, which has implications for nerve preservation, as injury to predictable nerve pathways can significantly impact sensation. Detailed anatomical studies have highlighted the importance of the ACBs and LCBs of the ICNs in NAC sensation. The LCB of the fourth ICN typically emerges at a consistent surface landmark, located ∼33 mm lateral to the pectoral border and 8 mm superior to the fifth rib^1^. Disruption of the anterior division of this nerve during mastectomy is strongly associated with the loss of NAC sensation^5^. Similarly, the lateral aspects of the ACBs of the third, fourth, and fifth ICNs run toward the NAC from a medial direction and contribute to sensation. Injury to these ACBs has been shown to significantly reduce breast sensation, underscoring their sensory importance^2^.

rNSM represents a paradigm shift in breast surgery, as it offers great precision, utilizing smaller, more lateral incisions, often to the axilla or inframammary fold, and avoids periareolar incisions, therefore potentially reducing nerve injury. Early clinical experience with rNSM has demonstrated both feasibility and oncological safety, as well as improvement in postoperative nipple sensation, likely due to the incision location and decreased tissue trauma^6^. A recent rNSM clinical series by Farr *et al*.^6^ reports preservation of up to 55% of nipple sensation after surgery, an outcome that is strikingly superior to traditional open nipple-sparing mastectomy approaches, which typically preserve nipple sensation in only ∼27—40% of cases^7,8^. Alongside robotic techniques, other innovations such as neurotization and nerve grafting have shown promise in restoring sensation post-mastectomy^9^. Comparisons of incision placement in breast procedures indicate that laterally positioned incisions, away from the NAC, result in better postoperative sensory outcomes^10^. A recent systematic review by Smeele *et al*.^2^ reinforced the predominance of the fourth ICN in NAC innervation, while also emphasizing the contributions of adjacent nerves and the need to spare these during surgery if possible. Seminal anatomical work provided early mapping of breast innervation, but did not fully capture the fine details regarding the variability or course of these nerves^11^. In light of emerging surgical techniques, a refined understanding of NAC sensory anatomy is needed, as preserving nipple sensitivity is increasingly recognized as essential for patient satisfaction and quality of life^12,13^.

This study leverages cadaveric dissections and a systematic literature review to delineate the precise anatomical pathways and variability of nerve branches innervating the NAC, and to translate these insights into actionable guidance for rNSM approaches.

## Methods

### Cadaveric dissection

This study examined six breasts from three female cadaveric donors from the Anatomical Gift Programme of the Department of Anatomy and Regenerative Medicine of the Royal College of Surgeons in Ireland (RCSI) University of Medicine and Health Sciences, Dublin. Prehumously, all donors had consented to the donation of their remains for the purposes of research. The study was approved by the Departmental Committee on Anatomical Examination and permission for the study was granted by the Institutional Licence Holder.

Donors were embalmed using a standardized protocol via arterial perfusion (with a venous drain) of a standard mix containing 6.25% formaldehyde (Formaldehyde 35%, Ocon Chemicals, CRTSF0130716), 12.5% glycerol (anhydrous, 99.0%–101%, Honeywell, CC15523), 3.125% phenol (90% aqueous solution, Panreac Applichem, CA141323.1611), and 78.125% methylated spirit (99% solution, TE Laboratories, CRTSI0330716) followed, if needed, by spot injections using the same fluid. After embalming, as per standard institutional protocol, the donors were stored at 4°C until transfer to the Anatomy Room for examination.

Dissections focused on tracing the lateral aspects of ACBs and the anterior aspects of LCBs to the NAC. Senior surgeons and anatomists provided expertise on dissections. Photographic documentation was conducted, yielding 66, 103, and 95 images from cadaver 1, cadaver 2, and cadaver 3 respectively. The dissection times were 35, 29, and 31 h for cadaver 1, cadaver 2, and cadaver 3 respectively.

### Data collection and illustrations

A total of 264 photographs were reviewed. Key nerves and their branches were annotated on these images. Composite illustrations to consolidate the findings were used to visualize the courses of the nerves innervating the NAC.

### Systematic literature review

Included studies described anatomical and clinical characteristics of NAC innervation, emphasizing nerve pathways relevant to breast sensation, rNSM implications, sensory-preservation outcomes, and embryological development of ICNs. Peer-reviewed clinical trials, anatomical dissections, systematic reviews, observational studies, and case reports were eligible for inclusion. Non-human studies or studies published outside of 1999–2024 for embryological studies and 2008–2024 for anatomical studies were excluded unless seminal. The literature search followed modified PRISMA 2020 guidelines for anatomical reviews (*Fig. S1*)^14^. Searches were conducted from 24 December 2024 to 28 March 2025, using PubMed and Embase databases. The following search terms were used: ‘nipple areolar complex’, ‘NAC’, ‘breast’, ‘innervation’, ‘cutaneous nerves’, ‘intercostal nerves’, ‘sensory nerves’, ‘anatomy’, ‘cadaveric study’, ‘nerve branches’, and ‘embryology of intercostal nerves’. Boolean operators (AND and OR) were used to refine searches. Filters used were: English, German, and Spanish language, human studies, female subjects, full-text availability, clinical studies, anatomical dissections, clinical trials, systematic reviews, RCTs, and meta-analyses. Additional articles recommended by senior surgeons and anatomists were included to ensure a comprehensive review of the available literature.

### Data extraction

Titles and abstracts were screened, followed by detailed full-text review. Articles were selected based on anatomical descriptions of NAC innervation, cadaveric or clinical data, emphasis on ICN contributions, and relevance to surgical implications. Extracted data focused on anatomy, sensory outcomes, and surgical relevance. Data were systematically synthesized into concise summaries, informing the anatomical insights and clinical recommendations presented in this study.

## Results

**Fig. 1:**
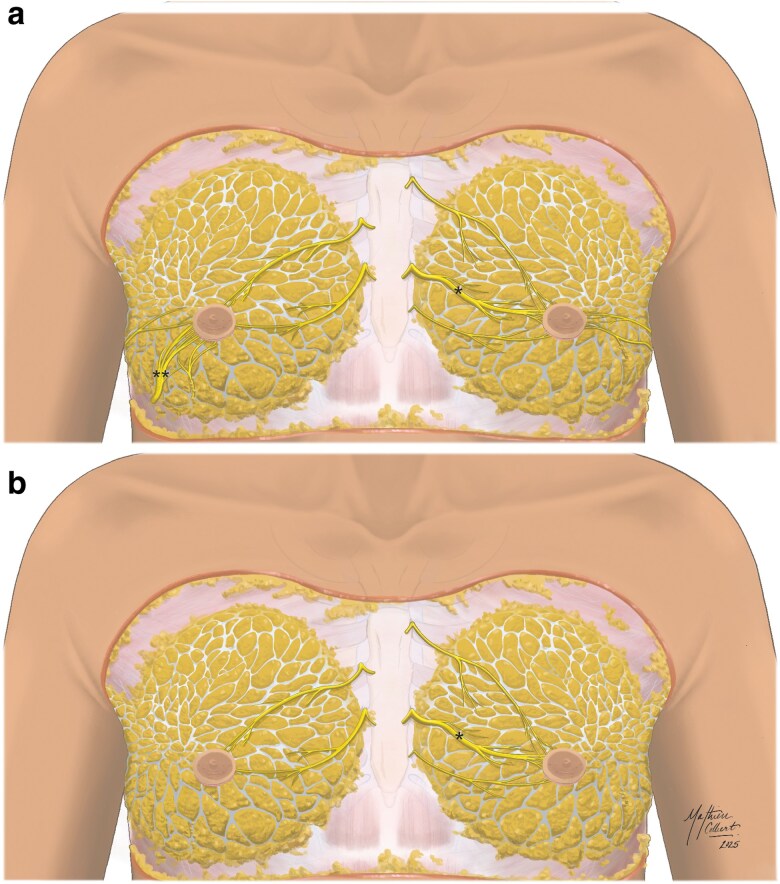
Composite illustrations demonstrating cadaveric NAC innervation **a** Both superficial ACBs and deeper LCBs supplying the NAC are shown, as identified across the six breasts. The calibre of each nerve is representative of the number of times the nerve was identified in this study. Nerves specific to right breasts and nerves specific to left breasts are shown. A single asterisk identifies the left fourth anterior cutaneous nerve (lateral branch), which was found in two of the three left breasts. A double asterisk identifies the right fifth lateral cutaneous nerve (anterior branch), which was found in two of the three right breasts. The remaining nerves that are shown were found once in their respective breasts. **b** Only the superficial ACBs are shown. NAC, nipple-areolar complex; ACBs, anterior cutaneous branches; LCBs, lateral cutaneous branches.

Cadaveric dissections of six breasts consistently identified the third, fourth, and fifth ICNs as being responsible for the primary innervation of the NAC. The fourth ICN emerged as the main nerve to the NAC across five of the six specimens, though significant variability was observed, with no two specimens demonstrating identical innervation. *Figure 1* highlights the trajectories of both the anterior and lateral branches of the relevant ICNs, and the ancillary branches, distinguishing superficial and deep pathways.

### Dominant contribution

**Fig. 2:**
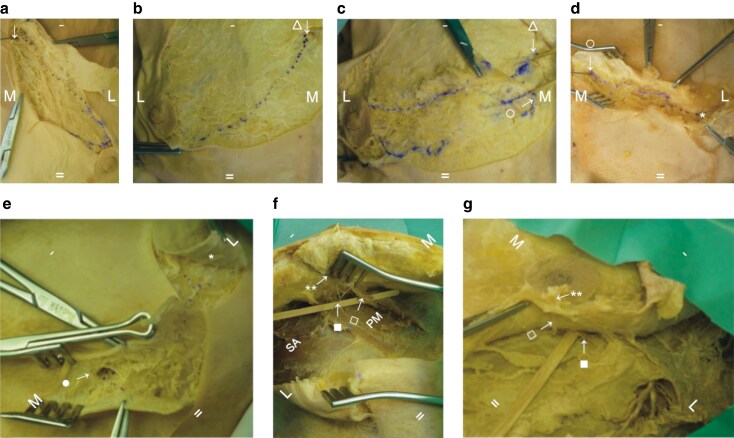
Cadaveric NAC innervation For orientation of images, - is cranial, = is caudal, M is medial, L is lateral, SA is serratus anterior, and PM is pectoralis major. **a** Second ICN emerging from parasternal intercostal space (downwards arrow), running alongside artery to the NAC. **b** Right third anterior cutaneous nerve (lateral branch) emerging from intercostal space (open triangle) and superficially traversing glandular tissue to the NAC. **c** Right fourth anterior cutaneous nerve (lateral branch) emerging from intercostal space (open circle), traversing momentarily inferior to right third anterior cutaneous nerve (resected and lifted to show path of the fourth) before reaching the NAC. **d** Left fourth anterior cutaneous nerve (lateral branch) (open circle) traversing superficially to the NAC (single asterisk). **e** Left fifth anterior cutaneous nerve (lateral branch) (filled circle) emerging from parasternal intercostal space before traversing superficially to the NAC (single asterisk). **f** Right fourth lateral cutaneous nerve (anterior branch) (filled square) running under glandular tissue, then rising to unite with fourth ancillary branch (open square) before both traversing through glandular tissue (double asterisk) to innervate the NAC. **g** Left fourth lateral cutaneous nerve (anterior branch) (filled square) running under glandular tissue, then rising to unite with fourth ancillary branch (open square) before both traversing through glandular tissue (double asterisk) to innervate the NAC. NAC, nipple-areolar complex; ICN, intercostal nerve.

The ACB of the fourth ICN consistently provided substantial innervation to the breast skin and NAC in each cadaver. Specifically, the lateral division of the fourth ICN ACB demonstrated a superficial course directly beneath the dermis, superficial to the breast tissue, travelling directly to innervate the NAC (*Fig. 2c*,*d*). Conversely, the anterior division of the LCB of the fourth ICN coursed more deeply, traversing through breast parenchyma toward the NAC. Two specimens exhibited the fourth ICN LCB anterior division ascending through breast tissue before curving toward the nipple (*Fig. 2f,g*). Thus, the fourth ICN provided both superficial and deep NAC innervation, via its ACB and LCB respectively.

### Supplementary contributions

Adjacent ICNs contributed variably to NAC innervation. The fifth ICN contributed superficially via its ACB lateral division (*Fig. 2e*). The third ICN also occasionally provided sensory innervation via its ACB. In one breast, the lateral division of the third ICN ACB travelled superficially toward the NAC, whereas its medial division terminated medially near the sternum (*Fig. 2c*). The second ICN contributed in a single instance, through its ACB lateral division, travelling alongside a blood vessel at the 4 o’clock position, terminating within the NAC (*Fig. 2a*).

### Ancillary nerve branches

Ancillary nerves, that is small nerve branches emerging from atypical locations, were identified in two of the three cadavers. In one specimen (*Fig. 2f*, an ancillary nerve branch originating from the fourth ICN emerged atypically, traversed beneath the breast tissue, and approached the underside of the NAC. In another specimen (*Fig. 2g*), an anterior ancillary branch was observed arising from the fourth ICN at an exit point lateral to the midclavicular line, travelling deeply underneath breast tissue toward the NAC, ultimately uniting with the anterior branch of the fourth ICN LCB before supplying the NAC.

### Superficial *versus* deep pathways

The lateral divisions of the ACBs, particularly from the third, fourth, and fifth ICNs, consistently displayed superficial trajectories immediately beneath the dermis and superficial to breast tissue. These nerves were located adherent to the superficial dermis, as well as within or superficial to the plane typically elevated as skin flaps during mastectomy. The anterior divisions of the LCBs consistently exhibited deeper, intraparenchymal pathways within the breast tissue, travelling less predictably toward the NAC. The fourth ICN LCB anterior division consistently demonstrated a deeper trajectory through breast glandular tissue.

## Discussion

Earlier studies recognize the fourth ICN as the predominant contributor to NAC sensation^2,15^, with this study providing a refined map of NAC innervation. Distinct anatomical pathways for NAC innervation have been demonstrated, distinguishing between superficial ACBs and deeper LCBs. Specifically, the ACBs run superficially beneath the dermis, making them targets for preservation through surgical techniques, while the deeper LCBs traverse glandular tissue, increasing their vulnerability during resection. Previously undocumented ancillary nerve branches were also identified, underscoring greater anatomical complexity than previously appreciated. These findings challenge conventional assumptions of uniform NAC innervation, suggesting the presence of sensory pathways for individualized surgical planning.

**Fig. 3:**
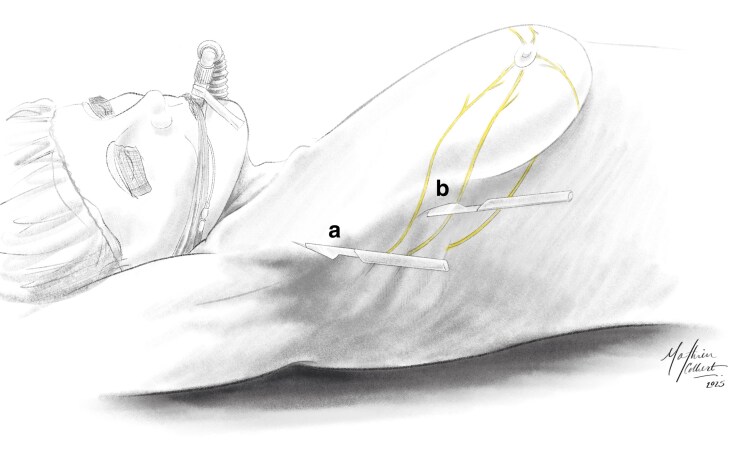
Anatomical line drawing demonstrating surgical approach strategies reported in rNSM Relevant ICNs, including both superficial ACBs and deeper LCBs, as found in this study, are depicted as emerging from the intercostal space and travelling to the nipple areolar complex. **a** The super-lateral approach taken by Farr *et al*.^6^ is shown. **b** The inferior-lateral approach taken by Toesca *et al*.^17^ is shown. rNSM, robotic-assisted nipple-sparing mastectomy; ICNs, intercostal nerves; ACBs, anterior cutaneous branches; LCBs, lateral cutaneous branches.

These anatomical findings have direct surgical implications, especially for rNSM. By delineating nerve pathways, this study proposes a practical, anatomically based framework for intraoperative nerve-preservation strategies. The results demonstrate that sensory preservation is an attainable goal, complementing traditional priorities such as oncological safety and reconstructive outcomes in breast surgery. This study underscores the emerging clinical imperative of preserving postoperative nipple innervation as a determinant of subsequent patient satisfaction and quality of life^12,13,16^. Precise anatomical data, particularly highlighting nerves critical for sensation, offer actionable insights to inform evolving surgical approaches that integrate sensory preservation with oncological management. The introduction of rNSM has provided opportunities to enhance nerve preservation due to its precision and lateral incision. The more lateral incisions of Farr *et al*.^6^ and Toesca *et al*.^17^ (*Fig. 3*) allow surgeons to dissect tissue from lateral to medial, avoiding direct incision near the NAC^18^. Such a lateral approach can facilitate the preservation of superficial nerve branches, particularly the ACBs of the third, fourth, and fifth ICNs, with the fourth ICN being the most critical. Specifically, the superficial course of these nerves from the parasternal region toward the NAC allows lateral surgical approaches to spare them by dissecting deep to their pathway.

Preserving superficial nerve branches, particularly the fourth ICN ACB, may thus substantially enhance postoperative sensory outcomes. However, due to their inherent anatomy, deeper LCBs remain at risk during mastectomy, but intact superficial innervation can maintain considerable NAC sensation. Early clinical evidence supports this hypothesis. Farr *et al*.^6^ recently reported that after rNSM of 40 breasts, postoperative sensation was preserved in 95% of breast skin areas and in 55% of NAC regions, markedly exceeding outcomes after conventional nipple-sparing mastectomy. These clinical findings align closely with the anatomical observations from the present study, indicating that lateral surgical approaches can effectively preserve critical superficial NAC nerves, thus providing a clear anatomical rationale for improved sensory outcomes observed in robotic techniques^6,17,19^.

Recent work by Soares Domingues Polita *et al*.^20^ demonstrated significant sensory dysfunction immediately after nipple-sparing mastectomy, with gradual recovery over 1 year; the importance of incision placement was highlighted, as sensory recovery was superior in medial quadrants compared with more lateral quadrants. Further supporting these advancements, Rancati *et al*.^21^ demonstrated successful preservation of NAC sensory function during traditional nipple-sparing mastectomy using fluorescence imaging to identify and protect the fifth ICN and its branches; their postoperative sensory assessments confirmed complete recovery of tactile and thermal sensation at 3 months, suggesting that this approach may significantly reduce nerve injuries. Given the variability of innervation demonstrated in this study, it may become imperative in the future to integrate fluorescence imaging into robotic surgical platforms, enabling real-time, intraoperative visualization and preservation of critical sensory nerves. Equipping robotic surgical systems with fluorescence technology could enhance surgical precision and further improve sensory outcomes after rNSM. Emerging techniques such as intraoperative nerve monitoring or mapping also hold promise to further refine nerve preservation during robotic-assisted mastectomies^21^. Integration of these technological advancements, informed by detailed anatomical maps, may help surgeons verify the functional integrity of critical nerves, before proceeding with resection. Furthermore, where resection of sensory nerves is oncologically necessary, recent evidence supports the effectiveness of reconstructing sensory nerves. Peled *et al*.^22^ demonstrated substantial improvements in postoperative NAC and breast sensation through nerve reconstruction (that is neurotization) during nipple-sparing mastectomy with implant-based reconstruction, with nerve-grafting techniques also shown to be effective^23^. This was also demonstrated in an early technical report in which sensate implant-based reconstruction was achieved by coapting the fourth ICN to the NAC using a processed nerve allograft.^24^

**Fig. 4:**
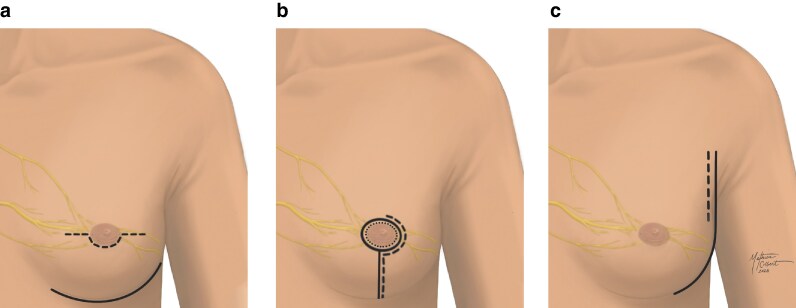
Composite illustrations demonstrating common surgical incision approaches for open nipple-sparing mastectomy in relation to underlying nerve anatomy **a** An omega incision (dashed line) and an inframammary fold incision (solid line). **b** A circumareolar incision (dotted line), a periareolar incision with vertical extension (dashed line), and a circumareolar incision with vertical extension (solid line). **c** A lateral incision (dashed lined) and a lateral incision with inframammary extension (solid line).

These anatomical findings carry implications for incision planning in breast surgery. The observed distinction between superficial and deep nerve pathways suggests that incision strategies should prioritize the preservation of superficial nerve branches wherever safe from an oncological perspective. This supports the use of lateral or inframammary incisions over radial or periareolar incisions, as these risk transecting the superficial ACB nerves. This is corroborated by clinical evidence, with less sensory loss using lateral and inframammary incisions than periareolar approaches^10,25^. Consequently, surgeons performing open nipple-sparing mastectomy may also benefit from adopting lateral incisions to improve sensory outcomes. *Figure 4* demonstrates common nipple-sparing mastectomy incisional approaches and their relativity to the underlying innervation. Understanding of NAC innervation has implications for modern breast surgery, especially as nerve-sparing and sensory-restoration techniques become standard components of reconstructive planning. Surgeons should identify and preserve the primary nerve branches innervating the NAC, specifically the fourth ICN lateral ACB and anterior LCB. Preoperative planning could be improved by using imaging techniques or marking known nerve exit points, such as the chest wall location of the fourth ICN branches^1^. Intraoperative approaches, including gentle subdermal flap dissection, fluorescent imaging, and nerve stimulation, could further protect nerve integrity.

This study has inherent limitations. Peripheral nerve anatomy was shown to have great variability, as has been demonstrated in similar papers assessing different anatomical regions such as the head and neck, thorax, and lower limbs. The small sample size of six breasts may not fully represent the full spectrum of variability of NAC innervation^26–30^ . Further studies with larger cohorts, complemented by high-resolution imaging such as MRI-neurography or intraoperative nerve mapping, such as that employed by Rancati *et al*.^21^, would be valuable to expand these findings. Additionally, the dissection times do not represent typical operating times for mastectomy. Dissecting embalmed tissue demands different techniques than used on live tissue; moreover, the aim of this study was to provide detailed anatomical insights, including photography, therefore extending the duration of procedures compared with surgical contexts.

## Supplementary Material

znaf232_Supplementary_Data

## Data Availability

Anonymized data will be available from the corresponding author upon reasonable request.
