# Supplementary figures and images for: Anatomical basis for sensory preservation in robotic mastectomy
Source: Br J Surg. 2025 Nov 18;112(11):znaf232. doi: 10.1093/bjs/znaf232 (PMC12624855; doi:10.1093/bjs/znaf232)

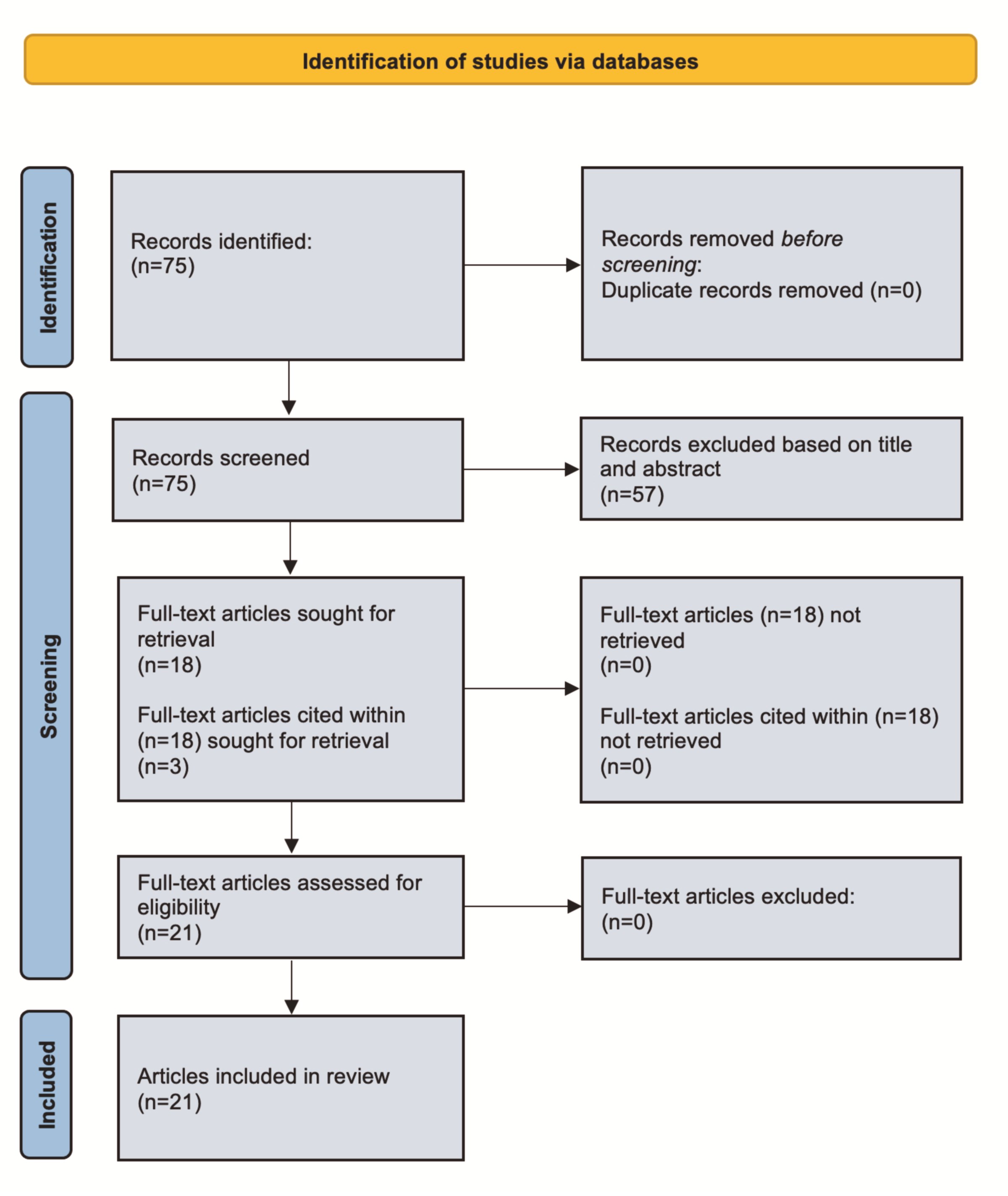

Supplement: znaf232_Supplementary_Data [file znaf232_supplementary_data.jpeg]
